# A scientometric analysis of research trends on emerging contaminants in the field of cancer in 2012–2021

**DOI:** 10.3389/fpubh.2022.1034585

**Published:** 2022-11-25

**Authors:** Daitian Zheng, Lingzhi Chen, Huiting Tian, Qiuping Yang, Jinyao Wu, Zeqi Ji, Jiehui Cai, Yexi Chen, Zhiyang Li

**Affiliations:** Department of General Surgery, The Second Affiliated Hospital of Shantou University Medical College, Shantou, China

**Keywords:** emerging contaminants, cancer, scientometric, Bibliometrix, CiteSpace, VOSviewer

## Abstract

**Introduction:**

Recently, emerging contaminants have been discovered in the aquatic environment that can cause a range of human diseases, including cancer. In this study, our scientometric analysis provides a comprehensive overview of emerging contaminants and cancer research from 2012 to 2021.

**Methods:**

The Web of Science Core Collection Database was used to retrieve all related publications. The bibliometix R-package, CiteSpace, and VOSviewer were applied to collect information on annual citations and publications, famous journals and authors, the most productive countries and organizations, popular topics, and keywords.

**Results:**

A total of 2378 publications were retrieved. The publication's output showed a gradual upward trend from 2012 to 2021. The most-cited paper was a review article by Vandenberg et al. that was published in 2012. According to the analysis results, the United States published the most articles. The closest collaboration was between the United States and China. *Environmental Research and Science of The Total Environment* published the most paper. It was Choi KC who was the most productive and had the highest h-index, g-index, and m-index among the authors. The most frequently used keywords were “exposure,” “endocrine-disrupting chemicals,” “endocrine disruptors,” “cancer,” “bisphenol-a,” and so on.

**Discussion:**

Emerging contaminants play a significant role in cancer development. However, most studies are conducted *in vivo* with human cells or animal models, and relatively few are on human models. The scientometric analysis offers researchers a clear picture of the current state of research and hotspots in this field. From our study, researchers may find some hotspots that merit in-depth investigation.

## Introduction

Cancer is commonly recognized as a disease in which some of the body's cells grow uncontrollably and destroy the healthy physiology of our bodies. According to an article written by H. Sung et al. on global cancer statistics, it was estimated that around 19.3 million new cancer cases and almost 10.0 million cancer-related deaths occurred in 2020. Female breast cancer is the most common type of cancer (11.7% of the total cases), followed by lung (11.4%), colorectal (10.0 %), prostate (7.3%), and stomach (5.6%) cancers. Lung cancer remains the leading cause of cancer deaths (18.0% of all cancer deaths), followed by colorectal (9.4%), liver (8.3%), stomach (7.7%), and female breast (6.9%) cancers (1). By 2040, it is estimated that there will be 28.4 million people diagnosed with cancer globally, a 47% increase from 2020 (2). The etiology of cancer is complex, including biological, chemical, physical, psychological, genetic, as well as environmental factors (3). Nevertheless, the etiology of cancer development is far from understood completely (4).

In recent years, a wide range of compounds of anthropogenic or natural origin have been discovered in the aquatic environment. There are various sources of these contaminants, ranging in concentration from ng L^−1^ to μg L^−1^ (5). These contaminants are called “emerging contaminants (ECs)” (6). A number of ECs are currently known, including endocrine disrupting chemicals (EDCs), pharmaceuticals and personal care products (PPCPs), microplastics, disinfection by-products (DBPs), perfluorinated compounds, organophosphate flame retardants (OPFRs), brominated flame retardants (BFRs), and so on (7–9). In addition to harming the environment, ECs pose a severe threat to human health as well (10–15). ECs can cause a wide range of diseases in humans. Mutagenic and carcinogenic effects are induced by some ECs, including the development of breast cancer and prostate cancer (16). Furthermore, according to a review article published in 2020, some other types of cancer are associated with EDCs, for instance, papillary thyroid cancer, testicular cancer, and kidney cancer (17). Other systematic reviews have listed many ECs as carcinogens, including OPFRs (18), bisphenol-A (BPA) (19, 20), and glyphosate (21). Research into emerging pollutants' effects on tumors is topical at the moment. However, no scientometric analysis has been conducted on the role of ECs in cancer research.

In this study, a scientometric analysis of the field was therefore conducted to give a broad overview of the current research situation. In this analysis, we aim to find out annual citations and publications, famous journals and authors, the most productive countries and organizations, popular topics, and keywords from the last 10 years.

## Methods

### Aim

In this study, we aimed to identify authors, journals, countries, and organizations that were most representative in the research of ECs in the field of cancer. The other aim was to get a clear understanding of the current state of this field's research and provide researchers with some enlightenment and research ideas.

### Design

From the Web of Science, we retrieved 2,378 publications related to ECs and cancer over the past decade. Next, by using Web of Science's “Analysis Results” and “Citation Report” functions, we obtained basic information about these 2,378 publications. Then, we imported the data into the CiteSpace software, VOSviewer software, as well as Biblioshiny website to conduct a scientometric analysis.

### Sample

We chose the Web of Science as a source of academic publications because it contains more articles and is more comprehensive than other databases like PubMed.

### Data acquisition

Web of Science (WoS) was used to retrieve all publications on ECs and cancer. Search options are determined: editions = “SCI-EXPANDED (2003–present),” and database = “Web of Science Core Collection.” As search strategies, “neoplasms,” “endocrine disruptors” and “microplastics” were used as medical subject headings (Mesh). In order to assure the quality of the data retrieved, a variety of topic keywords were used to retrieve the relevant publications. The searched content is as follows: #1, TS = “emerging contaminant^*^” OR TS = “contaminant^*^ of emerging concern” OR TS = “emerging pollutant^*^” OR TS = “personal care product^*^” OR TS = “pharmaceutical^*^ and personal care product^*^” OR TS = “endocrine disruptor^*^” OR TS = “endocrine disrupting chemical^*^” OR TS = “endocrine disrupting compound^*^” OR TS = “microplastic^*^” OR TS = “disinfection byproduct^*^” OR TS = “disinfection by-product^*^” OR TS = “perfluorinated compound^*^” OR TS = “perfluorochemical^*^” OR TS = “brominated flame retardant^*^” OR TS = “organophosphate flame retardant^*^”; #2, TS = “neoplasm^*^” OR TS = “malignancy” OR TS = “malignancies” OR TS = “neoplasia^*^” OR TS = “cancer^*^” OR TS = “tumor^*^”; #3, “#1,” and “#2.” Moreover, these publications were filtered to include only those published between 2012 and 2021. The search was conducted on August 26th, 2022, and produced 2,510 documents. Our next step was to select articles or reviews as the document type and English as the language, which resulted in 2,441 publications, including 1,929 articles and 512 reviews. A total of 69 publications were excluded: 1 news item, 1 correction, 6 letters, 20 editorial materials, 15 meeting abstracts, 8 non-English review papers, and 18 non-English papers. Then, after analyzing the gathered literature with Endnote, we discovered 10 early access publications and 53 articles published in 2022, which we then eliminated. Finally, a total of 2,378 publications were included in the scientometric study, including 501 review articles and 1,877 articles, which together accounted for 78.93% of the total.

### Data analysis and research tools

We preliminarily obtained information about the research fields, languages, document types, journals, publication years of these papers, authors, affiliations, countries, funding agencies, publishers, open access, and so on using the Web of Science's “Analyze Results” function. The “Citation Report” feature of the Web of Science also provided other information, including the h-index, the average citations per term (ACI), self-citation frequency for citing articles and citation frequency for all citing articles, the number of times cited without self-citations, as well as the total number of times cited.

The data was imported into the CiteSpace software, VOSviewer software, as well as Biblioshiny (a web interface for Bibliometrix), including document types, titles, abstracts, languages, authors, affiliations, cited references, and keywords.

The complete scientometric analysis was carried out using Bibliometrix, one of the important R packages in the R-studio (version 4.2.1), and the results were visualized with Bibliometrix images (22). By downloading the raw data of these publications and importing it into the Biblioshiny website, we obtained their basic and important information, including time span, the number of documents, the number of sources, authors, and authors' collaboration, the number of references, document contents, document types. With the information, we were able to make a quick assessment of whether the results fulfilled our standards. Aside from that, other information included the production of scientific research each year, the average number of articles cited per year, most cited documents and references, authors (authors' production over time, the most relevant authors, author influence ranked by m-index, g-index, and h-index), the contribution of countries and affiliations, sources (source dynamics, journal sources, and most relevant sources), and keywords. We used a three-field plot to summarize the relationship between the most prolific authors, the most productive countries, and the best-known organizations. A keyword plus co-occurrence network was used to assist in the detection of hotspot research. Keyword pairs with high co-occurrences have a high degree of correlation.

CiteSpace (version 6.1.R3) helped find the keywords with strong citation bursts, obtain citation bursts for references, and depict the dual-map of the citation relationship between journals (23, 24).

VOSviewer (version 1.6.18), as an excellent choice of network analysis software, was used to produce maps from network data, analyze the documents that were cited most often, create keyword co-occurrence network analysis, and a co-citation network analysis of references. Moreover, this software was applied to determine the strength of links between authors, affiliations, and countries in our study (25).

IBM SPSS statistics 26 was used to conduct the statistical analyses. Numerical variables, such as the average number of articles cited per year, were expressed as means plus/minus standard deviation and median with maximum and minimum values. Frequencies and percentages were used to express categorical variables. We used Spearman's correlation coefficient to confirm the statistical significance and check for any correlations between the selected variables. It was considered statistically significant if the *p*-value was < 0.05 (*p* < 0.05) in all tests.

**Figure 1:**
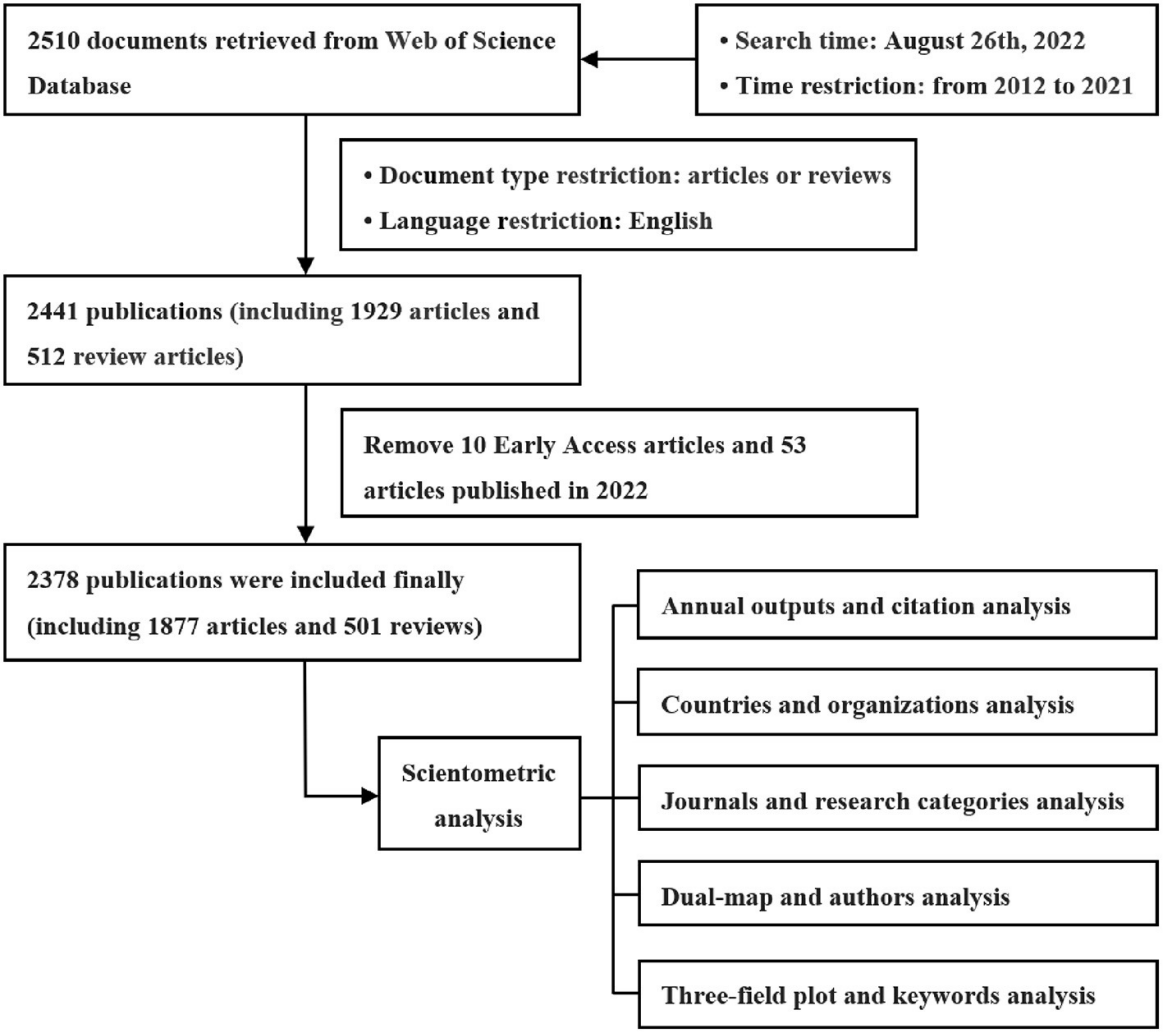
Flowchart of literature collection and analysis.

Figure 1 shows a step-by-step procedure for collecting literature and analyzing the results. This study used public data and there was no need to seek the approval of the ethics committee.

## Results

### Annual outputs

**Figure 2:**
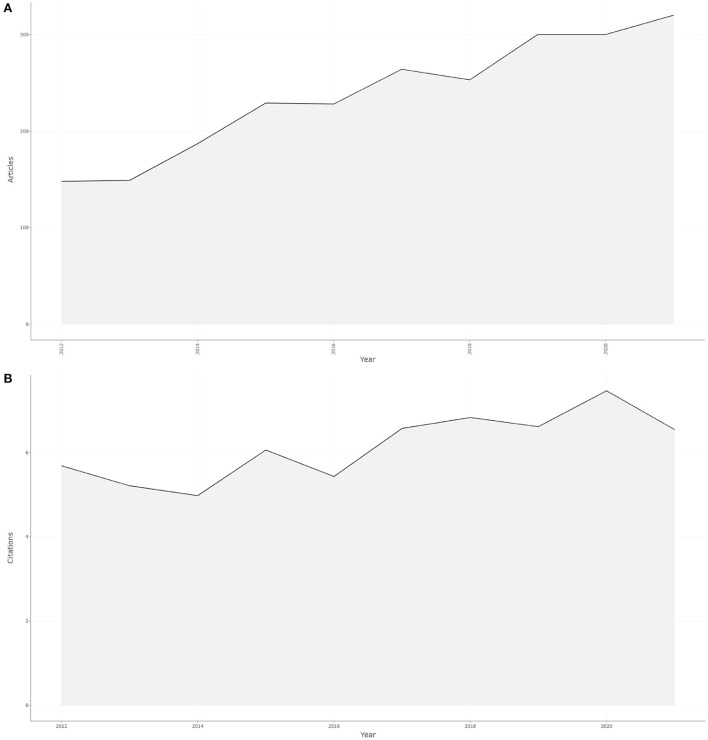
The trend of the publications output and the average article citations on the research of emerging contaminants and cancer from 2012 to 2021. **(A)** The trend of the publications output on the research of emerging contaminants and cancer from 2012 to 2021. **(B)** The trend of the average article citations on the research of emerging contaminants and cancer from 2012 to 2021.

From the Web of Science, we retrieved a total of 2,378 papers on this topic over the past 10 years. Overall, as shown in Figure 2A (r = 0.977; *p* < 0.001]), we can see a gradual increase trend from 148 papers (6.22%) published in 2012 to 320 papers (13.46%) published in 2021. A rapid period of development occurred from 2013 to 2015 and from 2016 to 2019. In terms of increasing annual scientific production, the median yearly growth rate was 8.95%, with a maximum of 25.50% in 2014.

### Citation analysis

The citation analysis is an impartial and simple method of evaluating research institutions, the quality of papers, journals, and even the performance of a researcher. The scientific impact of an article is determined by how many times it is cited (26). Out of all the papers that were retrieved 42,193 citing papers were found, including 1,602 self-citations (accounting for 3.80%) and 40,591 that were not (accounting for 96.20%). The total number of citations for the paper was 68,102, including 6,751 self-citation (accounting for 9.91%) and 61,351 without self-citation (accounting for 90.09%). There was an average of 28.64 times cited per document. In Figure 2B, article citations each year from 2012 to 2021 show a fluctuating pattern, with the highest number in 2020 (7.47).

Using VOSviewer, we can create Supplementary Table S1, which gives detailed information about the top 10 papers in the area of ECs and cancer research. By analyzing the papers that were highly cited, we could identify the areas of research that were currently at the fore-front. All of these studies were published between 2012 and 2020. Published in 2012, a review article written by Vandenberg et al., which had 1,804 citations, is the most frequently cited paper (27). The article “EDC-2: The Endocrine Society's Second Scientific Statement on Endocrine-Disrupting Chemicals” by Gore, A. C. et al. (published in 2015) received 1,135 immediate citations (28). The third-ranked article was “Bisphenol A–Sources, toxicity and biotransformation” written by Michalowicz (2014) (cited 536 times) (29). A total of 4 studies were cited more than 500 times, accounting for 0.17% of all cited articles; 15 papers (0.63%) received more than 300 citations, and 107 (4.50%), more than 100. The top-cited work (27) received 980 citations over the past 5 years, accounting for 54.32 percent of all its number of citations, while the second-most-cited paper (28) received 949 citations (83.61% of all its number of citations).

**Figure 3:**
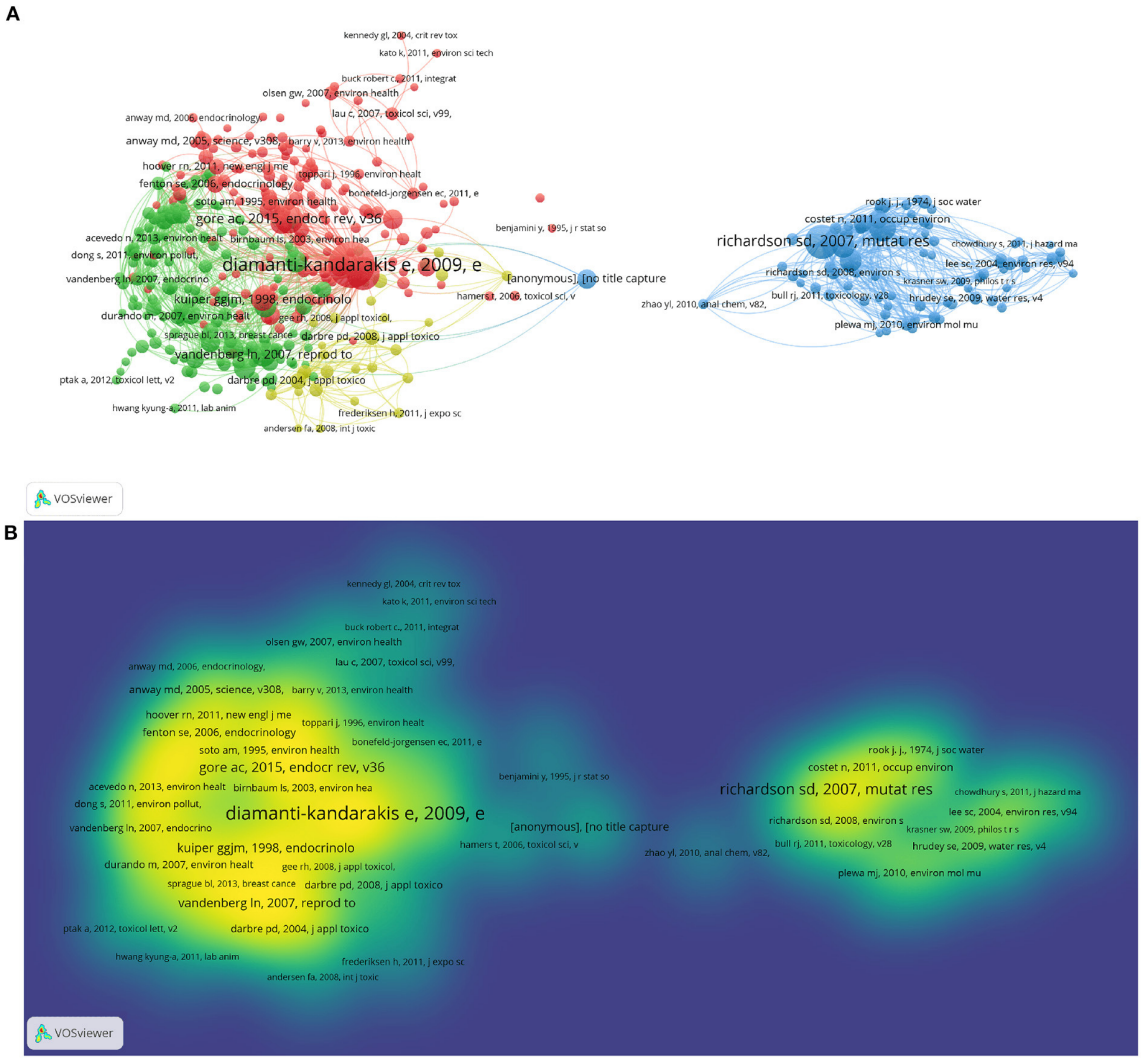
The network map of co-citation between references with 20 or more 20 citations. **(A)** The network visualization map of co-citation between references with 20 or more 20 citations. **(B)** The density visualization map of co-citation between references with 20 or more 20 citations. The degree of color is proportional to the number of co-citations (green: low citations; yellow: high citations).

Figure 3 was conducted by VOSviewer, which depicted a co-cited map of these articles' references and helped us explore changes connected to the important clusters of publications. There were 397 references that met our criteria, as defined by the minimal requirement of 20 citations for a cited reference. Nodes grow in size as more references are cited (Figure 3A). The degree of color corresponds to the number of co-citations (green: low citations, yellow: high citations) (Figure 3B). In Figure 3A, cited references are grouped into four clusters. The cluster with the largest area has 155 items, shown in red, indicating the most attractive research area. In Figure 3, the paper with the highest frequency of citations (288) is the one written by Diamanti-Kandarakis, E (2009), representing its significance in this research field (30). The 5 most cited references in order were from Diamanti-Kandarakis, E (2009) (288 citations) (30), Richardson, SD (2007) (181 citations) (31), Vandenberg, LN (2012) (172 citations) (27), Gore, AC (2015) (135 citations) (28), and Villanueva, CM (2004) (122 citations) (32). We identified the top 25 references with the highest citation bursts in timeline distribution with the help of CiteSpace (Supplementary Figure S1). It is worth noting that among these references, seven references began to experience a citation burst in 2019, six in 2013, and four in 2012. Additionally, a total of 12 references were continuously cited up to 2021. Published in 2009, the work by Diamanti-Kandarakis, E, with citation bursts from 2012 to 2014, was the one that had the strongest (strength = 31.25) citation burst (30).

### Countries, organizations, and journals analysis

#### Countries analysis

**Figure 4:**
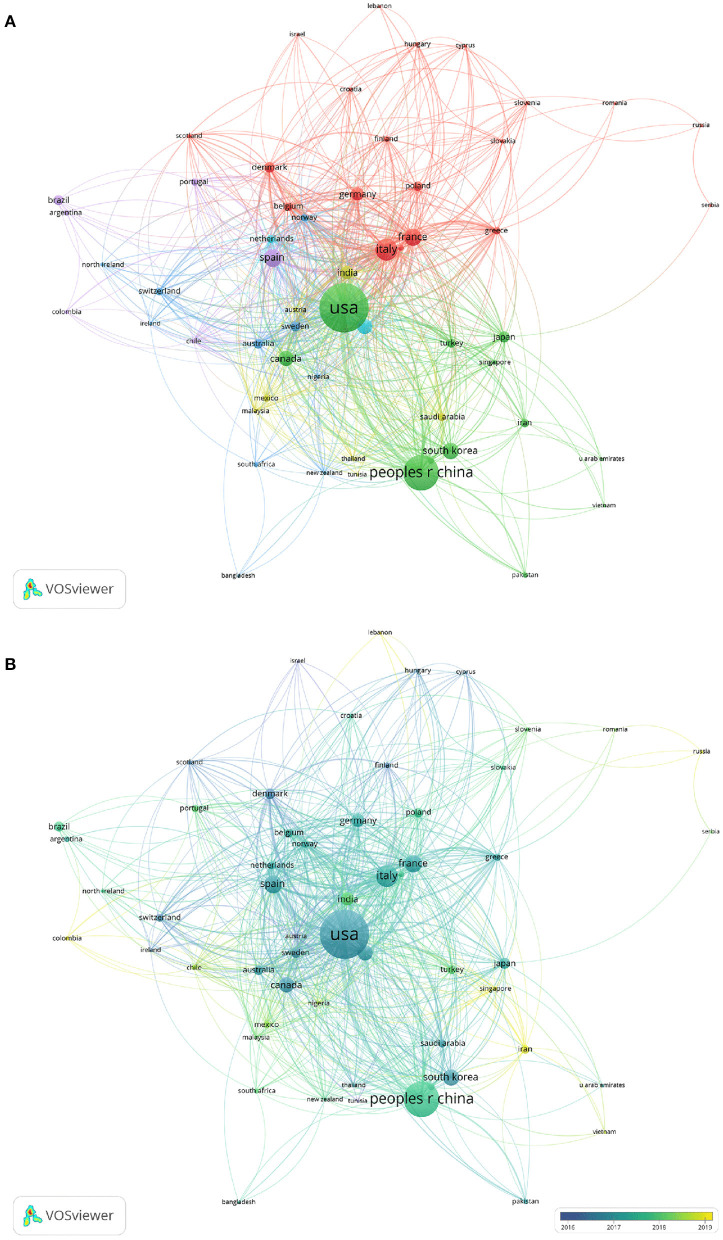
The network map of co-authorship between countries with more than five publications. **(A)** The network visualization map of co-authorship between countries with more than five publications. **(B)** The overlay visualization map of co-authorship between countries with more than five publications. The distance between circles reflects the relationship strength between different countries based on how frequently they occur together. The size of the circle represents their total link strength.

A total of 2,378 documents were contributed by 95 countries or regions to this field of research. Among them, developed countries account for the majority, and there are also some developing countries. Most of the studies related to the topic were published in the USA, which was the most productive country in this regard (*n* = 626, accounting for 26.32% of all). China came after the United States (*n* = 431, 18.12%). They were followed by Italy (*n* = 137, 5.76%), Korea (*n* = 111, 4.67%), Spain (*n* = 92, 3.87%), and France (*n* = 92, 3.87%). Table 1 lists the top 10 most cited countries. Articles published in the United States had the most citations (*n* = 24191), and then China ranked second (*n* = 8788) and was followed by Italy (*n* = 4230), Canada (*n* = 2826), and Spain (*n* = 2630). The co-authorship analysis included 58 nations with over five publications in this field of study. Among the top 5 nations in order of total link strength, the USA came in the first place (total link strength = 496), followed by Italy (268), England (257), Spain (252), and France (233) (Figure 4). Circles' distance from each other reflects the relationship strength between various nations based on how frequently they occur together, and the circle's size represents their total link strength. Over time, some undeveloped countries started to play an increasingly important role in this research field. Among them, China ranks first with a total link strength of 203, and India comes in second (total link strength = 88). They were followed by Saudi Arabia (strength = 73) and Turkey (strength = 66). In Table 2, the cooperation among countries/regions was obtained using the bibliometrix package. The two countries that cooperated most closely are the United States and China.

**Table 1:** Top 10 cited countries contributing to this research area.

**Country**	**Total production**	**Total citations**	**Average article citations**
USA	626	24191	38.64
China	431	8788	20.39
Italy	137	4230	30.88
Canada	64	2826	44.16
Spain	92	2630	28.59
France	92	2393	26.01
Korea	111	2287	20.60
Denmark	36	1981	55.03
India	66	1899	28.77
United Kingdom	47	1869	39.77

**Table 2:** The cooperation among countries/regions.

**From**	**To**	**Frequency**
USA	China	66
USA	Canada	40
USA	United Kingdom	38
USA	Spain	34
USA	Italy	33
Spain	Italy	29
China	Canada	23
Italy	United Kingdom	23
Spain	United Kingdom	23
USA	France	23

#### Organizations analysis

**Figure 5:**
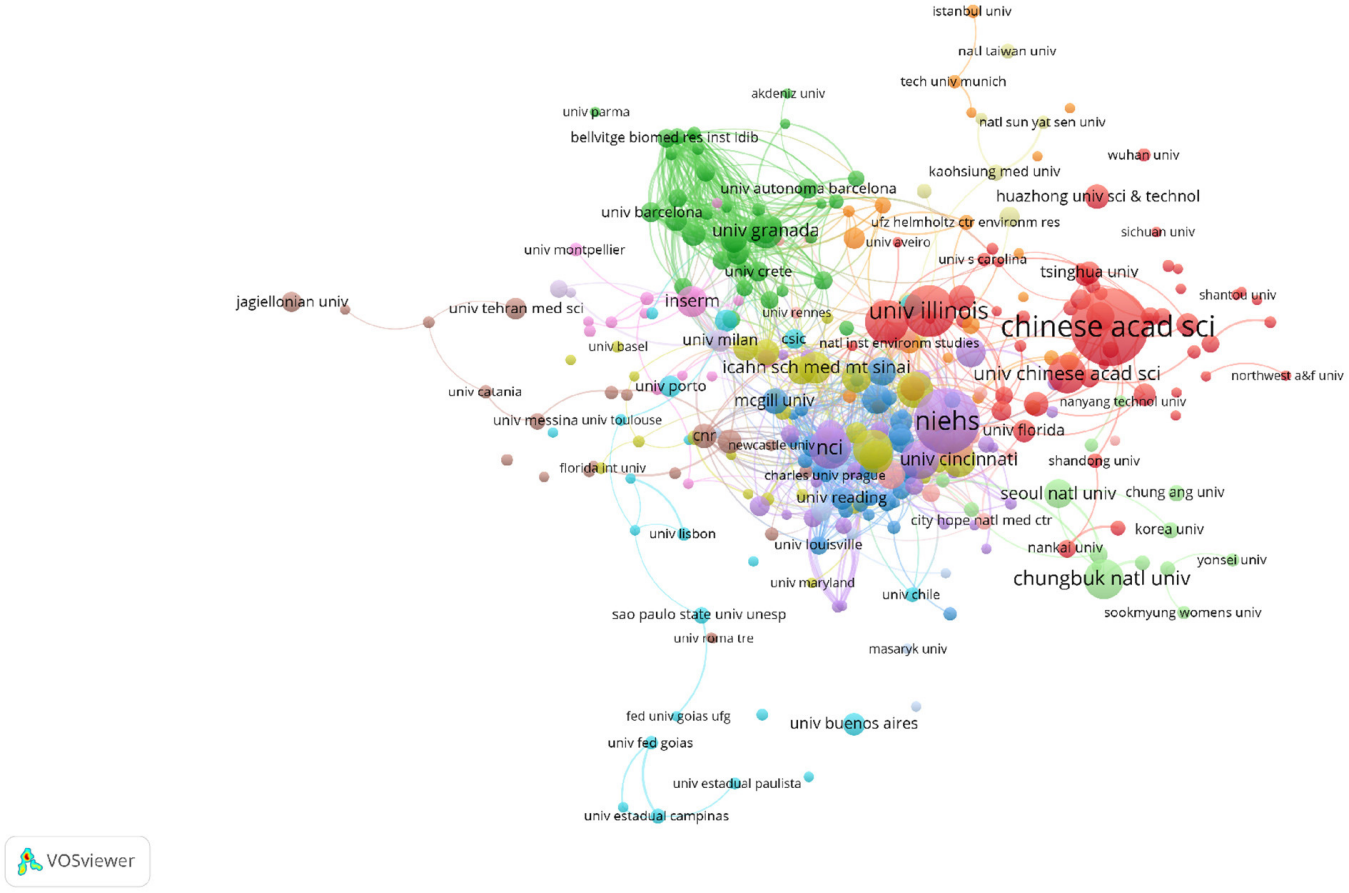
The network visualization map of co-authorship between organizations with more than five publications.

A total of 2,904 organizations have been involved in this research area. The number of publications from the Chinese Academy of Sciences was the highest (*n* = 82, 3.45%). The organization is located in China and following it was the National Institute of Environmental Health Sciences (*n* = 65, 2.73%), the University of Illinois System (*n* = 46, 1.93%), the United States Environmental Protection Agency (*n* = 36, 1.51%), and the National Cancer Institute (*n* = 34, 1.43%) (Table 3, Figure 5). Next, we looked at co-authorship for institutions with over five publications, resulting in 319 results. In order of the total link strength, the National Institute of Environmental Health Sciences (153), the National Cancer Institute (148), the University of North Carolina (138), the University of Oviedo (117), and the Center for Research in Environmental Epidemiology (111) were the top five organizations.

**Table 3:** Most productive organizations contributing to this research field.

**Organization**	**Total production**	**Total citations**	**Total link strength**
Chinese Academy of Sciences	82	1904	86
National Institute of Environmental Health Sciences	65	5228	153
University of Illinois System	46	3263	66
United States Environmental Protection Agency	36	1738	75
National Cancer Institute	34	1555	148
Chungbuk National University	32	1098	15
Centers for Disease Control and Prevention	31	917	83
University of California, Berkeley	30	3099	106
University of Massachusetts	30	4198	91
University of Chinese Academy of Sciences	30	442	46

#### Journals analysis

These 2,378 papers were published in 635 types of journals. In Supplementary Table S2, we listed the top 10 journals in this field according to productivity. A sum of 597 papers was published by the top 10 journals, which is 25.11% of all publications. *Environmental Research* and *Science of The Total Environment* published the most document (*n* = 79). The journal with 68 publications, *Environment International*, was the second most productive. Ranked No. 3, *Environmental Science and Technology* has 65 publications and was followed by *Chemosphere* (*n* = 61) and *Environmental Science and Pollution Research* (*n* = 52). The main focus of these journals is the environment. *Environment International* had the most citations with 3,203 and was followed by *Endocrine Reviews* (2993 citations), *Environmental Science and Technology* (2690 citations), *Environmental Research* (2376 citations), and *Science of The Total Environment* (2174 citations). As for the impact of the source, the h-index, developed by Hirsch (2005) (33), was commonly used to describe the significance of the journals (34). The journal with the highest h-index, *Environmental Science and Technology*, had a score of 29. It was followed by *Environment International* (h-index = 28), *Environmental Research* (h-index = 28), *Environmental Health Perspectives* (h-index = 27), *PLOS One* (h-index = 23), and *Science of The Total Environment* (h-index = 23) (Table 4). Over the years, *Chemosphere, Environment International, Environmental Research, Environmental Science and Technology, Environmental Science and Pollution Research* and *Science of The Total Environment* have remained active in this research area (Supplementary Figure S2).

**Table 4:** Source impact of the top 10 journals publishing in this area.

**Source**	**h-index**	**g-index**	**m-index**	**TC**	**NP**	**PY-start**
Environmental science & technology	29	51	2.636	2690	65	2012
Environment international	28	56	2.545	3203	68	2012
Environmental research	28	46	2.545	2376	77	2012
Environmental health perspectives	27	42	2.455	1813	47	2012
PLoS One	23	37	2.091	1453	45	2012
Science of the total environment	23	44	2.091	2174	79	2012
Chemosphere	19	32	1.727	1252	61	2012
Reproductive toxicology	19	34	1.727	1253	44	2012
Toxicological sciences	19	31	1.727	1015	37	2012
Environmental pollution	18	39	1.636	1620	50	2012

TC, total citations; NP, number of publications; PY-start, publication year start.

### Research categories analysis

The study involved 114 different research categories in all. Table 5 shows the top 5 subject categories by the number of publications. Published 921 articles, “Environmental Science” ranked first, followed by “Toxicology” (584 publications), “Public Environmental Occupational Health” (321 publications), “Endocrinology Metabolism” (192 publications), and “Engineering Environmental” (173 publications). According to the number of publications, the most well-known publishers were “Elsevier” (896 publications), “Springer Nature” (292 publications), “Wiley” (170 publications), “Mdpi” (148 publications), and “Amer Chemical Soc” (108 publications).

**Table 5:** Top 5 active research subject categories.

**Research subject categories**	**Documents**
Environmental Sciences	921
Toxicology	584
Public Environmental Occupational Health	321
Endocrinology Metabolism	192
Engineering Environmental	173

### Dual-map overlay analysis

**Figure 6:**
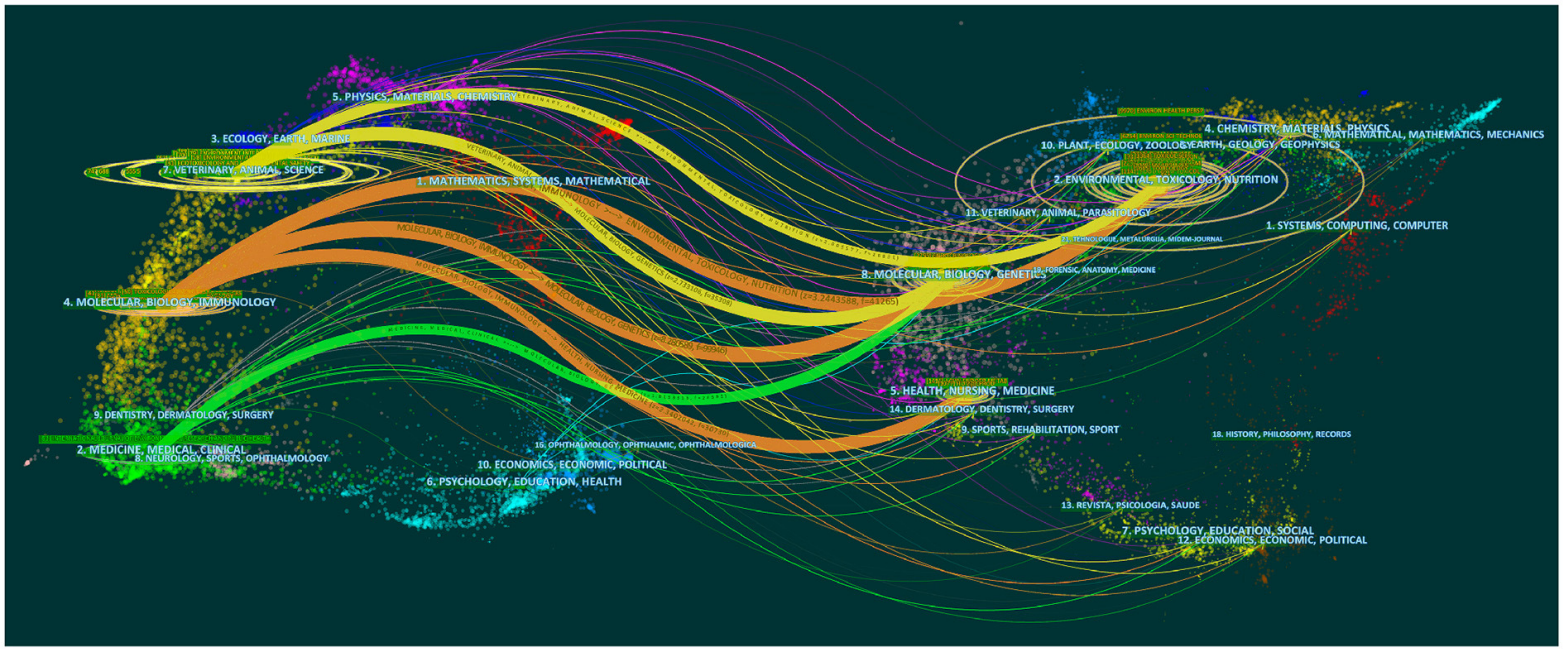
Dual-map overlay of journals publishing work related to ECs and cancer (citing journals are on the **left** and cited journals are on the **right**).

Dual-mapping analysis, designed by Chen and Leydesdorff, in which characteristics of publication portfolios can be analyzed, compared, and contrasted visually (35). It provides insight into the interdisciplinary nature of the journal and reveals its type or focus, which is widely used in bibliometrics (36, 37). In Figure 6, The dual-map of journals is shown using the CiteSpace (version 6.1.R2) program. Through the dual-map, researchers can have a clear view of the citation tracks and disciplines. In Figure 6, citing journals are on the left, and cited journals are on the right. They are connected by colored curves, which represent paths of references (38). As shown in Figure 6, the disciplines of “molecular, biology, immunology,” “veterinary, animal, science” and “medicine, medical, clinical” are the main sources of the cited journals. The citing journals come from a variety of disciplines, including “molecular, biology, genetics,” “environmental, toxicology, nutrition,” and “health, nursing, medicine”. Therefore, it can be concluded that research into ECs and cancer presents an interdisciplinary trend.

### Authors analysis

A total of 10,339 authors participated in our study and contributed to these 2,378 papers. Among them, 60 were single-authored document authors and 10,279 were multi-author document authors. There were 73 single-authored documents. Each article had an average number of co-authors of 6.24. The most prolific author was Choi KC, who contributed to 30 publications, which was 1.26% of all the publications. Villanueva CM was next with 27 publications, accounting for 1.14%. They were followed by Kogevinas M (*n* = 24, 1.01%), Hwang KA (*n* = 22, 0.93%), and Plewa MJ (*n* = 19, 0.80%) (Table 6). An author was more prolific, indicating that he/she was more important and representative in this area of research. Four authors have written more than 20 papers, while forty-four have written more than 10. An annual total of citations is represented by the intensity of the color, and the number of publications is represented by the size of the circle, as shown in Supplementary Figure S3. Articles by Choi and Hwang were primarily published in the first seven years, however, Villanueva and Kogevinas generally maintained a consistent trend.

**Table 6:** Top 10 contributing authors in the field of ECs and cancer.

**Authors**	**Articles**	**Articles fractionalized**	**h-index**	**g-index**	**m-index**	**TC**	**NP**	**PY-start**
Choi KC	30	7.36	22	30	2.000	1083	30	2012
Villanueva CM	27	2.42	15	27	1.364	1128	27	2012
Kogevinas M	24	1.45	14	24	1.273	723	24	2012
Hwang KA	22	4.91	19	22	1.727	844	22	2012
Plewa MJ	19	4.45	14	19	1.273	1163	19	2012
Li XF	18	3.72	13	18	1.182	1017	18	2012
Chen J	17	1.94	13	17	1.625	416	17	2015
Taboga SR	16	2.77	8	11	0.727	128	13	2012
Vandenberg LN	16	4.31	11	16	1.000	2940	16	2012
Zhang X	16	2.40	8	16	0.727	345	16	2012

TC, total citations; NP, number of publications; PY-start, publication year start.

In Supplementary Table S3, the top 5 authors are listed according to the frequency with which the publication was cited. With 2940 citations, Vandenberg LN ranked first. He was followed by Soto AM (2660 citations), Colborn T, Myers JP, Vom Saal FS, and Welshons WV (2332 citations), Heindel JJ (2242 citations), and Zoeller RT (2235 citations). According to the h-index, Choi KC was in the first place with an h-index of 22, Hwang KA was in second place (h-index = 19), and Villanueva CM was in third place (h-index = 15). They were followed by Kogevinas M and Plewa MJ (h-index = 14), and Chen J (h-index = 13). A total of twenty-six authors had an h-index over 10 among the most prolific authors. Taking into account the different ages of different scientists, the m-index as a correction of the h-index for time aids in identifying scientists who have truly succeeded (34). In order of m-index, Choi KC ranked first with an m-index of 2.000, followed by Hwang KA (1.727), Salamanca-Fernandez E (1.667), Chen J (1.625), Dirven H and Fantke P (1.500). An analysis was then conducted on 198 authors with five or more articles. As for link strength, Villanueva CM had the highest total link strength (770), Choi KC was ranked second (total link strength = 689), and Kogevinas M was ranked third (total link strength = 655). Following them were Hwang KA and Plewa MJ, with their total link strength of 554 and 527, respectively.

**Figure 7:**
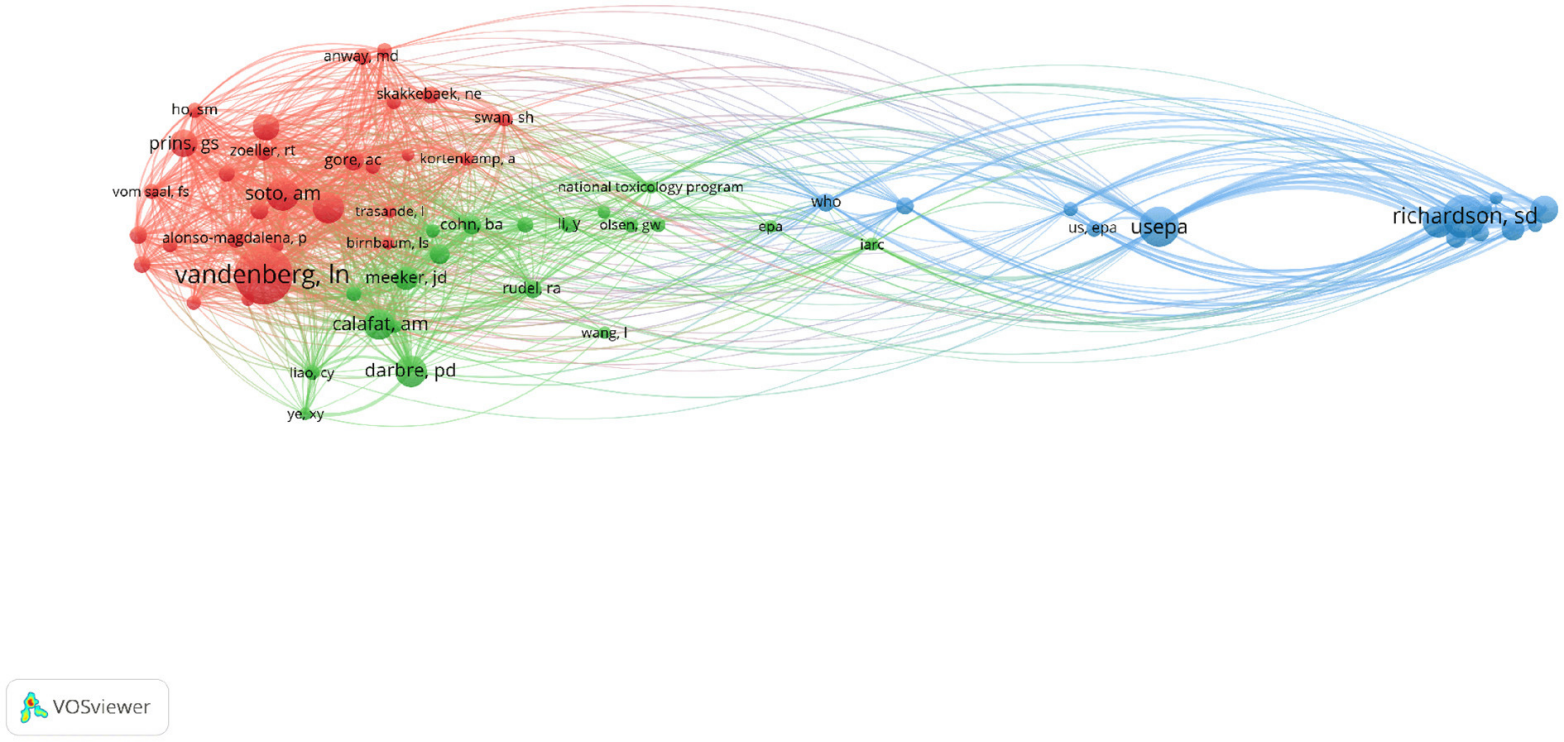
The network visualization map of co-citations between authors with 100 or more than 100 citations.

Detailed information on co-citation between authors is shown in Figure 7, which was created using VOSviewer. On the basis of a minimum requirement of 100 citations, a co-citation analysis of 62 authors was conducted. Vandenberg LN, who had a total link strength of 7736, came in top place in terms of link strength. With a total link strength of 4136, Soto AM came in second. Richardson SD, who came in third place (3823), Prins GS (3292), and Calafat AM were the next in line (2942).

### Three-field plot

A three-field plot is shown in Supplementary Figure S4, in which we can understand the relationships among the top 20 most productive authors (left), countries (middle), and organizations (right). The height of the nodes indicates the contribution of an author, organization, and country, while the thickness of the lines shows how many connections there are between them. The United States, China, and Spain were the countries with the most connections. The organization with the largest contribution in America was Emory University, followed by Columbia University. The top 3 authors, Kogevinas M, Villanueva CM, and Gracia-Lavedan E, had the strongest trend in collaboration across countries.

### Keywords analysis

Using VOSviewer software, 492 keywords were extracted from the 2,378 articles with more than 10 occurrences. Following that, the co-occurrence network was divided into various colored clusters by VOSviewer through its clustering function. The more relevant the keywords are, the more probable it is that they will be clustered together. As seen in the visual network map, all of these keywords can be categorized into six groups (Supplementary Figure S5A). In this way, it is possible to find out what research is currently being conducted. The keywords in Supplementary Figure S5C showed the same frequency on the visual density map. It is clear from the map that the keywords used most frequently were “exposure” (*n* = 417), “endocrine-disrupting chemicals” (*n* = 380), “endocrine disruptors” (*n* = 332), “cancer” (*n* = 307), “bisphenol-a” (*n* = 303), and so on. In addition, in Supplementary Figure S5B, the gradations of color show how frequently these keywords appear on average in respective articles. Blue indicates that these keywords appear early, while yellow later. Over time, the focus of this field of study shifted from the relationship between endocrine disruptors and breast cancer to the relationship between DBPs and bladder cancer, and then came the risk assessment and the removal of ECs.

Keywords plus, are words or phrases that are automatically generated by a computer algorithm and they appear frequently in the titles of an article's references, but not necessarily in the title itself (39, 40). There are four quadrants in Supplementary Figure S6 where different words or phrases are presented: (a) Motor themes (first quadrant): there are a number of words in this cluster that are highly established and crucial in this field of study due to their high centrality and density. (b) Niche themes (second quadrant): in spite of their development, they have become marginal in the overall field. (c) Emerging or declining themes (third quadrant): a low density and centrality characterize these themes, and they are poorly developed and related. There are two possibilities, one is that these themes are just emerging, and the other is that they are in decline. (d) Basic themes (fourth quadrant): these are themes with low density (underdeveloped) but high centrality (important). In this field of research, these are the fundamental concepts and knowledge. The keywords plus were clustered into four groups of four different colors each. As shown in Supplementary Figure S6, in this area of research, group 1's (red color) keywords (“endocrine-disrupting chemicals,” “bisphenol-a,” “breast-cancer”) were in the motor quadrant. However, group 2's (purple color) keywords (“polychlorinated-biphenyls,” “brominated flame retardants,” “persistent organic pollutants”) were in the second quadrant. Group 3's (green color) keywords (“drinking-water,” “disinfection by-products,” “bladder-cancer”) were in the third quadrant, indicating that they were undeveloped. Moreover, group 4's (blue color) keywords (“exposure,” “cancer,” “risk”) were located in the fourth quadrant, indicating that they were undeveloped but important in this field.

We used CiteSpace to find burst keywords, which refer to a word that is frequently cited in a specific period and represent research frontiers over time. The keywords with strong citation bursts were explored through CiteSpace, and the top 25 keywords were listed in Supplementary Figure S7. From 2012 to 2015, the leading seven keywords cited in the outbreak indicate the growing trend of ECs and cancer research. From 2013 to 2019, the middle twelve keywords, such as “receptor alpha,” “environmental estrogen,” and “epithelial cell” became active. A lot of attention has been paid to the following six keywords during the last few years, suggesting that they are the hotspots currently being researched. Those keywords with citation bursts keeping to 2021 (“polychlorinated biphenyls pcb,” “organophosphate flame retardant,” “contamination,” “metabolism,” “dbp,” and “endocrine”) may be the hottest topics. The keyword “breast cancer cell” had the most citation bursts (strength = 7.72) from 2012 to 2013.

## Discussion

A scientometric analysis of the publications related to ECs and cancer published between 2012 and 2021 was conducted using Bibliometrix, CiteSpace, and VOSviewer. There were 2378 documents on this topic up to the end of 2021. Between 2012 and 2021, the publication's output increased, peaking in 2021. Publication output grew by 8.95% on an annual basis, with the highest growth rate occurring in 2014, indicating that there was a sudden spike in interest in this area of research. The total number of citations for the paper was 68,102, with an average of 28.64 citations per document. The top 10 cited papers reflected the most recent findings and summarized the existing conclusions on the association between ECs and cancer. Among them, four are review articles on the link between the prostate and breast cancer and ECs (27–29, 41). In 2012, Vandenberg et al. published an important review article that has received a large number of citations. Undoubtedly, his work is a milestone in this field of study. Two articles published in “Endocrine Reviews” indicated that EDCs exposure influences breast cancer and prostate cancer incidence (27, 28). In the past few decades, evidence has accumulated that EDCs may adversely affect breast development and cancer susceptibility. EDCs can impair female reproductive tissue development in various ways, making it more sensitive to subsequent assaults from environmental chemicals and hormones (28). Other ECs have also been linked to increased cancer risk. An article published in the field of environment shows that drinking water contaminated with perfluorooctanoic acid (PFOA) is associated with an increased risk of cancer (42). In addition, glyphosate is now authoritatively classified as a probable human carcinogen by the World Health Organization's International Agency for Research on Cancer (43). Tris(2,3-dibromopropyl) phosphate (TDBPP), or brominated “Tris,” and chlorinated OPFRs were also listed as carcinogens in another article (44).

In our study, 95 countries contributed to this area of research in total. The top three countries were the USA, China, and Italy. It should be noted that among all countries, the United States had the largest number of link strength with other countries, publications, and citations. China is the only developing country in the top 5, demonstrating the limitation of developing countries in terms of research capability. It is important for developing countries to learn from developed countries, formulate their own strategies, and promote research progress in this area. The United States is home to four of the top five most productive organizations in this field. Additionally, all of the top 5 institutions based on link strength ranking are also located in the United States. It is without a doubt that the United States was the country that collaborated and contributed the most with other organizations and countries. Even so, researchers from some developing countries were very encouraged by the growing contributions from these countries in this field.

The study included 635 journals and 9 of the top 10 journals were classified as “Environmental Science” while the remaining 1 was listed as “Reproductive Biology.” *Chemosphere, Environment International, Environmental Research, Environmental Science and Technology, Environmental Science and Pollution Research* and *Science of The Total Environment* continue to produce high-quality articles in this area, which indicates the importance of environmental science categories.

There were 10,339 authors who contributed to these 2,378 papers in our study. Choi KC (*n* = 30, 1.26%), Villanueva CM (*n* = 27, 1.14%), Kogevinas M (*n* = 24, 1.01%), Hwang KA (*n* = 22, 0.93%), and Plewa MJ (*n* = 19, 0.80%) were the top five authors when it came to output in this research area. Given that Choi KC had the highest m-index, g-index, and h-index, there is no denying that he is a successful researcher in this research area. With the highest link strength among all authors, Villanueva CM concluded that long-term nitrate exposure in drinking water at levels below the European regulatory limit is positively correlated with colorectal cancer risk, and the risk of rectal cancer was increased by dietary nitrate from animal sources, and then he added that when nitrate is ingested under conditions that increase the formation of N-nitroso compounds, specific cancer risks may be increased (45, 46). His 2018 review article was the most frequently cited among his articles, with 378 citations. He made outstanding contributions to this field of research, helping others to gain a deeper understanding of how DBPs affect the development of cancer.

A total of 492 keywords with more than 10 occurrences were used in the studies published on ECs research in the cancer field between 2012 and 2021. Based on cluster analysis of co-occurrence keywords, this field of research was divided into six clusters. Clusters 1–2 consisted of more than 100 keywords. Cluster 1 was the largest, consisting of 135 keywords, mainly focused on the relationship between the effect of BPA on breast cancer. It has been suggested that BPA, used extensively in plastic manufacturing, may contribute to breast cancer development (47–49). Therefore, exposure to BPA should be restricted to help reduce the risk of breast cancer. At present, many studies show that there is a direct link between exposure to environmental doses of BPA and a high incidence of breast cancer *in vivo* studies with human cells and animal models. However, there are relatively few studies related to human models, so *in vivo* analysis is necessary to determine the effect of BPA on domestic or work-related exposure (47). The 101 keywords in Cluster 2, are mainly about the relationship between DBPs in drinking water and bladder cancer. Water disinfection can result in undesirable by-products. The most common DBPs, trihalomethanes (THMs), are widespread in drinking water (50–52). To show the associations between exposure to THMs in drinking water and bladder cancer risk, different studies have been conducted, such as epidemiological studies, large-scale cohort studies, case-control studies, etc. Cluster 4 consisted of 83 keywords, mainly focusing on the relationship between endocrine disruptors and breast cancer. Many EDCs are man-made chemicals that are widely used by the chemical, agricultural, cosmetic, and food industries, and they are still part of everyday life for many people (53, 54). Strong evidence has been provided that dioxins, diethylstilbestrol, dichlorodiphenyltrichloroethane, and BPA are associated with increased cancer risk. It is foreseeable that the assessment of the carcinogenicity of other EDCs is likely to be a hot topic in this area. From the keyword analysis, in the beginning, the main focus of this research area was on the relationship of EDCs to breast cancer. The researchers then shifted their attention to the relationship between DBPs and bladder cancer. Most recently, risk assessment and removal of ECs have become hot topics.

As far as we know, a scientometric analysis of ECs research in the field of cancer has never been conducted before. To identify research hotspots and collaborations between authors, organizations, and countries in this area, we used three different visualization tools.

However, there are some noteworthy limitations to our study. First of all, the study focused mostly on English journals, resulting in a lack of representation from other languages. Consequently, we cannot guarantee that the findings of our research will apply to studies published in other languages (55). Secondly, because it is difficult for scientometric software to directly merge results from several databases, we only used Web of Science (WoS) during the search and did not merge the results with those from PubMed, Scopus, Embase, and other search databases. It is worth noting that some journals in certain disciplines are not covered by the WoS. However, for scientometric studies, the most frequently used literature database is the Web of Science. It provides some primary analysis functions, such as “create citation report” and “analyze results,” making it the easiest and the most convenient tool to use. What's more, one may encounter the problem of unavailability of complete information about an article, such as the country of affiliation of the author. Searching manually for the author can avoid this problem (56). Screening articles is somewhat subjective, so it is necessary to obtain relevant criteria or a comprehensive opinion from many experts before screening an article. There are also some shortcomings in the software we used to analyze the results. For instance, the articles with the most citations may cover a variety of disciplines, leading to low specificity for our research area. Moreover, only the first author is considered in the co-citation analysis by VOSviewer, while none of the other authors are included. The date of publication of some articles may be ages ago, so their conclusions may be outdated, and not reflective of the current research findings. In our study, we did not include articles published after January 2022. It is our hope, however, that future researchers will be able to gain some enlightenment and research ideas from our study.

## Conclusion

A scientometric analysis of the retrieved papers published between 2012 and 2021 in the field of ECs and cancer is conducted in this article. Since 2013, the number of publications related to this field has grown rapidly. The United States and China are at the fore-front of ECs and cancer research and the cooperation between these two countries is relatively close. Three of the most productive journals are *Environmental Research, Science of The Total Environment*, and *Environment International*. Among the authors, Choi KC is the most productive and has the highest m-index, g-index, and h-index, and Villanueva CM has the highest link strength. Studying the mechanism of EDCs, BPA, and DBPs in the occurrence and development of cancer will be beneficial to the prevention and treatment of the disease. To solve the issue of the impact of ECs on cancer development, researchers need to strengthen their research. For example, we should conduct more studies related to human models to determine the effect of ECs on exposure at home or work. Given today's heavy cancer burden, ECs will continue to receive increased attention. In future studies, risk assessment and the elimination of ECs will be critical subjects.

## Data availability statement

The original contributions presented in the study are included in the article/Supplementary material, further inquiries can be directed to the corresponding authors.

## Author contributions

DZ, LC, and HT: conceptualization, data curation, methodology, software, formal analysis, investigation, visualization, and writing—original draft. QY, JW, and ZJ: methodology, visualization, investigation, and writing—review and editing. JC: supervision and writing—review and editing. YC and ZL: funding acquisition, resources, and project administration. All authors contributed to the article and approved the submitted version.

## Funding

This work was supported by the Special Fund Project of Guangdong Science and Technology (Grant Numbers: 210728156901524 and 210728156901519), Medical Scientific Research Foundation of Guangdong Province, China (Grant Number: A2021432), Shantou Medical Science and Technology Planning Project (Grant Numbers: 210521236491457, 210625106490696, 220518116490772, and 220518116490933), and Administration of Traditional Chinese Medicine of Guangdong Province project (Grant Number: 202205092315428030).

## Conflict of interest

The authors declare that the research was conducted in the absence of any commercial or financial relationships that could be construed as a potential conflict of interest.

## Publisher's note

All claims expressed in this article are solely those of the authors and do not necessarily represent those of their affiliated organizations, or those of the publisher, the editors and the reviewers. Any product that may be evaluated in this article, or claim that may be made by its manufacturer, is not guaranteed or endorsed by the publisher.
