# Supplementary material for: A scientometric analysis of research trends on emerging contaminants in the field of cancer in 2012–2021
Source: Front Public Health. 2022 Nov 25;10:1034585. doi: 10.3389/fpubh.2022.1034585 (PMC9733951; doi:10.3389/fpubh.2022.1034585)
Supplement: Supplementary file 1 [file Table_1.DOCX]

Supplementary Material

**Supplementary Table S1** Top 10-cited papers in the field of emerging contaminants and cancer with authors, sources, publication year, total citations, and total citations per year.

| Rank | Title | Author* | Source | Year | TC | TC per year |
| --- | --- | --- | --- | --- | --- | --- |
| 1 | Hormones and Endocrine-Disrupting Chemicals: Low-Dose Effects and Nonmonotonic Dose Responses | Vandenberg, Laura N.; Colborn, Theo; Hayes, Tyrone B.; Myers, John Peterson | ENDOCRINE REVIEWS,33(3),378-455 | 2012 | 1804 | 164 |
| 2 | EDC-2: The Endocrine Society's Second Scientific Statement on Endocrine-Disrupting Chemicals | Gore, A. C.; Chappell, V. A.; Fenton, S. E.; Zoeller, R. T. | ENDOCRINE REVIEWS,36(6),e1-e150 | 2015 | 1135 | 141.88 |
| 3 | Bisphenol A - Sources, toxicity and biotransformation | Michalowicz, Jaromir | ENVIRONMENTAL TOXICOLOGY AND PHARMACOLOGY,37(2),738-758 | 2014 | 536 | 59.56 |
| 4 | A review of the pathways of human exposure to poly- and perfluoroalkyl substances (PFASs) and present understanding of health effects | Sunderland, Elsie M.; Hu, Xindi C.; Dassuncao, Clifton; Allen, Joseph G. | JOURNAL OF EXPOSURE SCIENCE AND ENVIRONMENTAL EPIDEMIOLOGY,29(2),131-147 | 2019 | 517 | 129.25 |
| 5 | Environmental impact of estrogens on human, animal and plant life: A critical review | Adeel, Muhammad; Song, Xiaoming; Wang, Yuanyuan; Yang, Yuesuo | ENVIRONMENT INTERNATIONAL,99,107-119 | 2017 | 450 | 75 |
| 6 | Male Reproductive Disorders and Fertility Trends: Influences of Environment and Genetic Susceptibility | Skakkebaek, Niels E.; Rajpert-De Meyts, Ewa; Louis, Germaine M. Buck; Eisenberg, Juul, Anders | PHYSIOLOGICAL REVIEWS,96(1),55-97 | 2016 | 435 | 62.14 |
| 7 | Concerns over use of glyphosate-based herbicides and risks associated with exposures: a consensus statement | Myers, John Peterson; Antoniou, Michael N.; Blumberg, Bruce; Benbrook, Charles M. | ENVIRONMENTAL HEALTH,15 | 2016 | 409 | 58.43 |
| 8 | Parabens. From environmental studies to human health | Bledzka, Dorota; Gromadzinska, Jolanta; Wasowicz, Wojciech | ENVIRONMENT INTERNATIONAL,67,27-42 | 2014 | 408 | 45.33 |
| 9 | Environmental exposure to microplastics: An overview on possible human health effects | Prata, Joana Correia; da Costa, Joao P.; Lopes, Isabel; Rocha-Santos, Teresa | SCIENCE OF THE TOTAL ENVIRONMENT,702 | 2020 | 388 | 129.33 |
| 10 | Drinking Water Nitrate and Human Health: An Updated Review | Ward, Mary H.; Jones, Rena R.; Brender, Jean D.; van Breda, Simone G. | INTERNATIONAL JOURNAL OF ENVIRONMENTAL RESEARCH AND PUBLIC HEALTH, 15(7) | 2018 | 378 | 75.6 |

^*^ First, second, third, and last authors

**Supplementary Table S2** A) Top 10 popular journals regarding the number of related articles and B) top 10 cited journals regarding the number of citations.

1. Top 10 popular journals

| Journals | Articles | 2022 impact factor | 2022 JCR partition |
| --- | --- | --- | --- |
| ENVIRONMENTAL RESEARCH | 79 | 8.431 | Q1 |
| SCIENCE OF THE TOTAL ENVIRONMENT | 79 | 10.753 | Q1 |
| ENVIRONMENT INTERNATIONAL | 68 | 13.352 | Q1 |
| ENVIRONMENTAL SCIENCE & TECHNOLOGY | 65 | 11.357 | Q1 |
| CHEMOSPHERE | 61 | 8.943 | Q1 |
| ENVIRONMENTAL SCIENCE AND POLLUTION RESEARCH | 52 | 5.19 | Q2 |
| INTERNATIONAL JOURNAL OF ENVIRONMENTAL RESEARCH AND PUBLIC HEALTH | 50 | 4.614 | Q2 |
|  |  |  |  |
| ENVIRONMENTAL POLLUTION | 50 | 9.988 | Q1 |
| ENVIRONMENTAL HEALTH PERSPECTIVES | 47 | 11.035 | Q1 |
| REPRODUCTIVE TOXICOLOGY | 46 | 3.421 | Q2 |

1. Top 10 cited journals

| Cited journals | Citations | 2022 impact factor | 2022 JCR partition |
| --- | --- | --- | --- |
| ENVIRONMENT INTERNATIONAL | 3203 | 13.352 | Q1 |
| ENDOCRINE REVIEWS | 2993 | 25.261 | Q1 |
| ENVIRONMENTAL SCIENCE & TECHNOLOGY | 2690 | 11.357 | Q1 |
| ENVIRONMENTAL RESEARCH | 2376 | 8.431 | Q1 |
| SCIENCE OF THE TOTAL ENVIRONMENT | 2174 | 10.753 | Q1 |
| ENVIRONMENTAL HEALTH PERSPECTIVES | 1813 | 11.035 | Q1 |
| ENVIRONMENTAL POLLUTION | 1620 | 9.988 | Q1 |
| PLOS ONE | 1453 | 3.752 | Q2 |
| INTERNATIONAL JOURNAL OF ENVIRONMENTAL RESEARCH AND PUBLIC HEALTH | 1364 | 4.614 | Q2 |
| ENVIRONMENTAL HEALTH | 1310 | 7.123 | Q1 |

**Supplementary Table S3** Top 5 authors with the most times cited of their publications in the field of emerging contaminants and cancer.

| Author | Documents | Citations | Total link strength |
| --- | --- | --- | --- |
| Vandenberg LN | 16 | 2940 | 1229 |
| Soto AM | 10 | 2660 | 1256 |
| Colborn T | 3 | 2332 | 522 |
| Myers JP | 3 | 2332 | 522 |
| Vom Saal FS | 3 | 2332 | 522 |
| Welshons WV | 3 | 2332 | 522 |
| Heindel JJ | 6 | 2242 | 584 |
| Zoeller RT | 7 | 2235 | 654 |


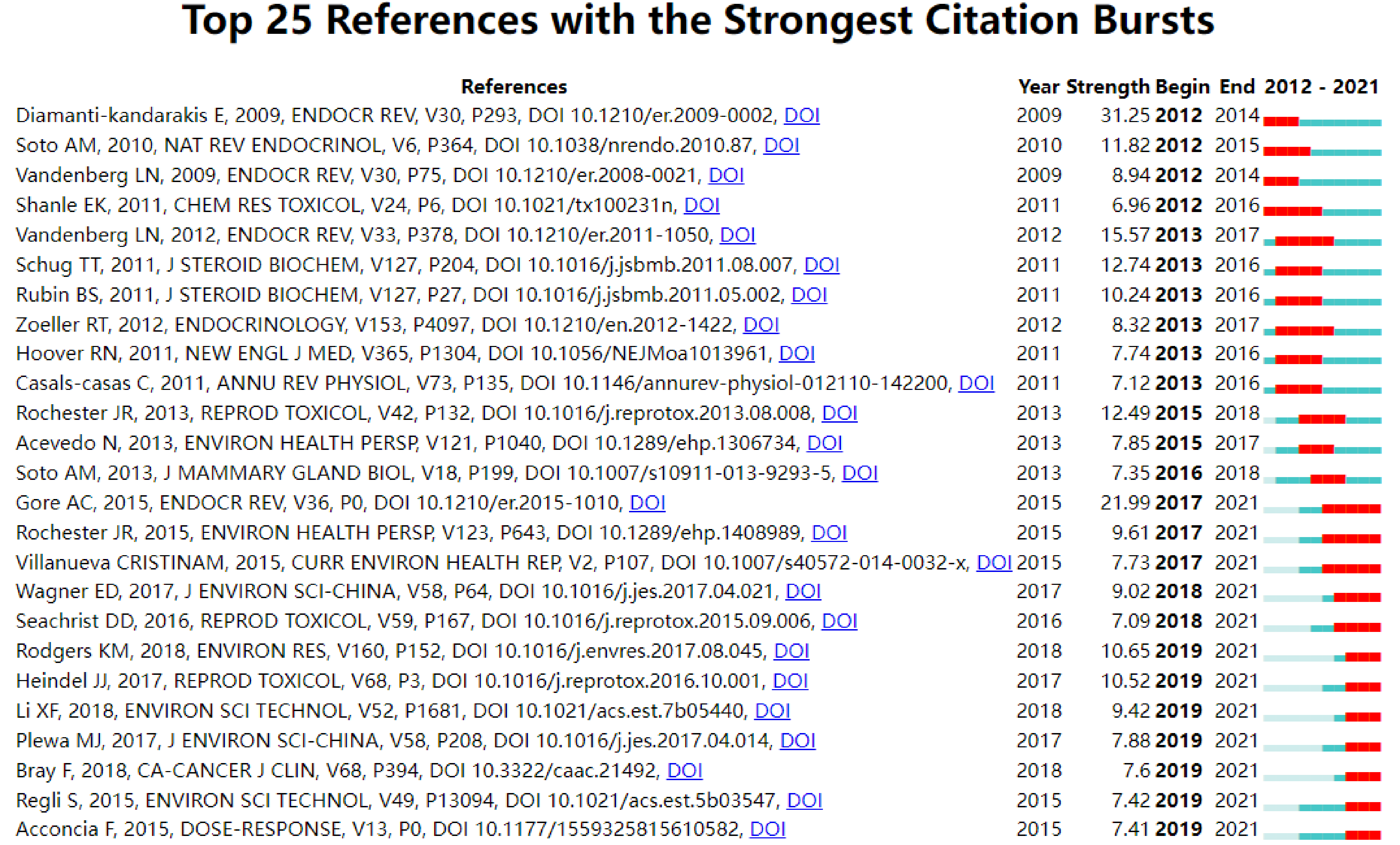


**Supplementary Figure S1** Top 25 references with the strongest citations burst.


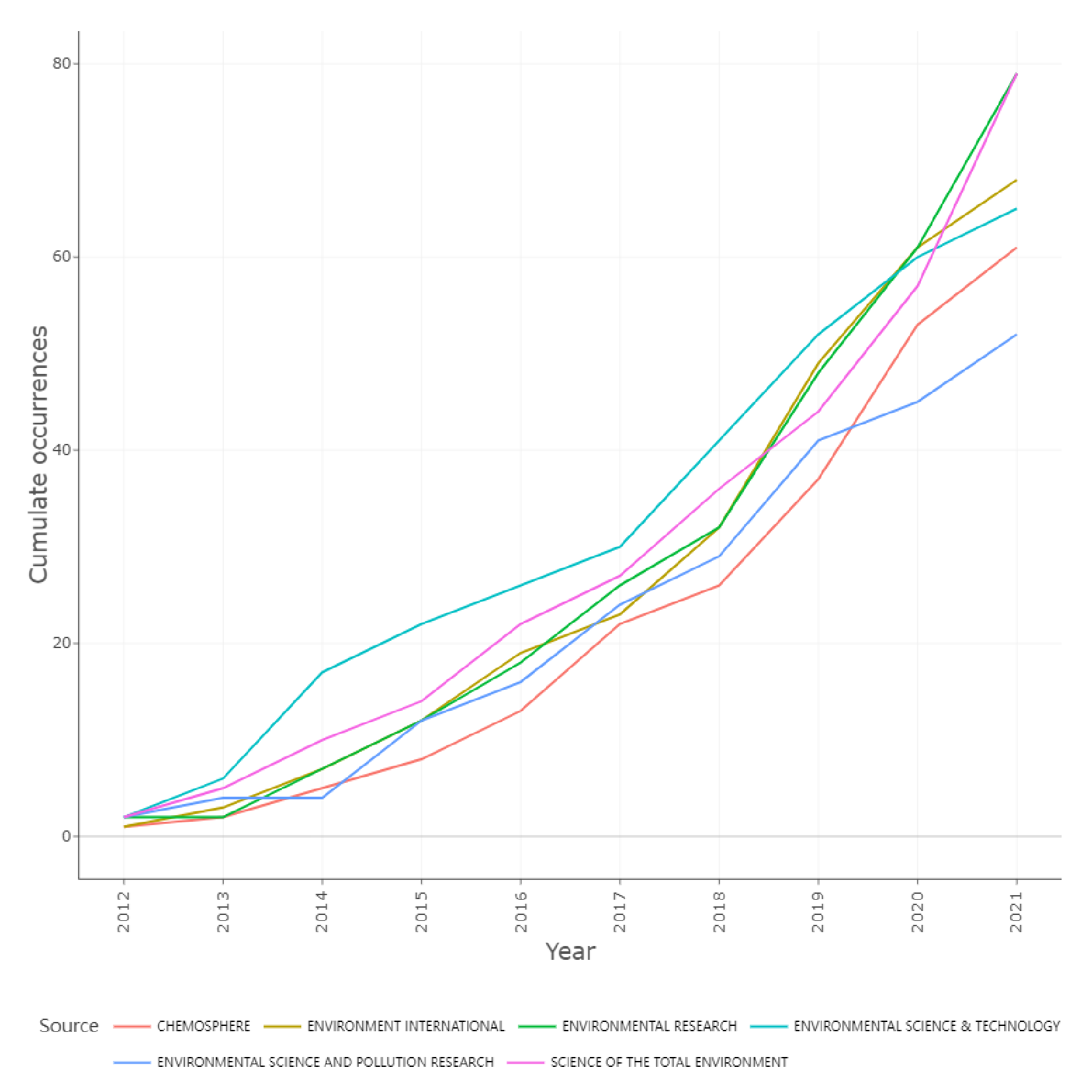


**Supplementary Figure S2** Source growth over the years related to this field of research.


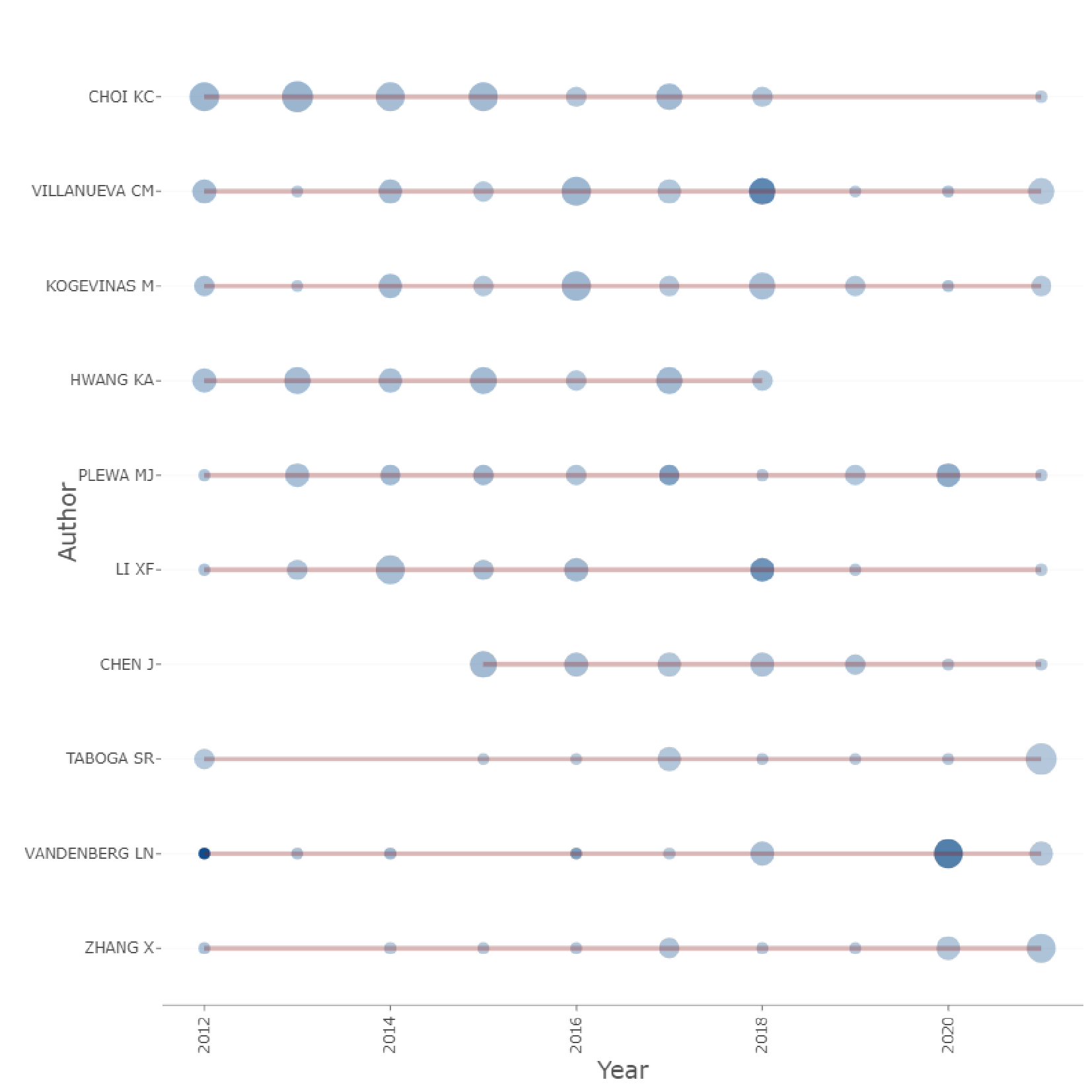


**Supplementary Figure S3** The number of author publications in the field over time.


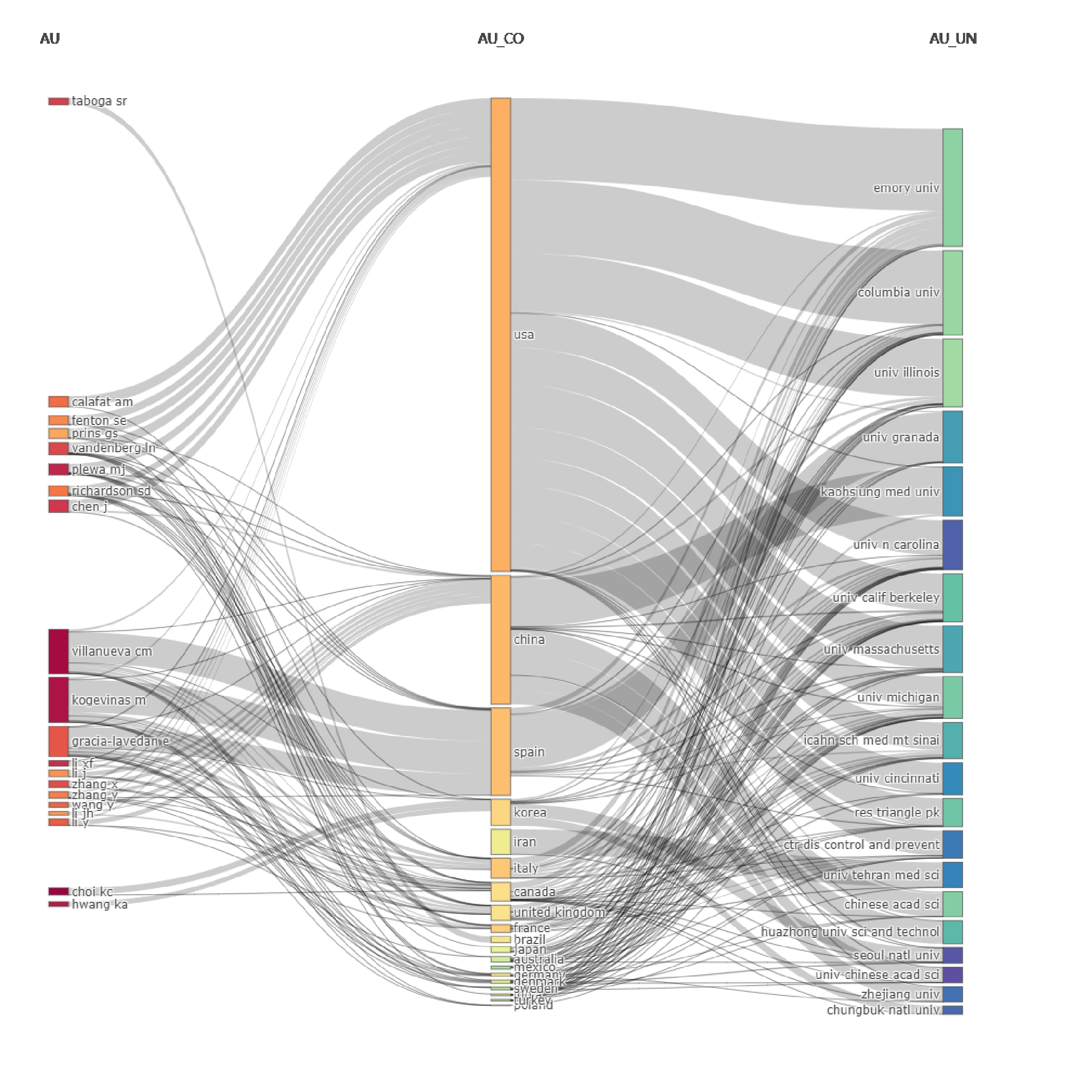


**Supplementary Figure S4** Three-field plot among the top 20 most productive authors (left), countries (middle), and organizations (right).


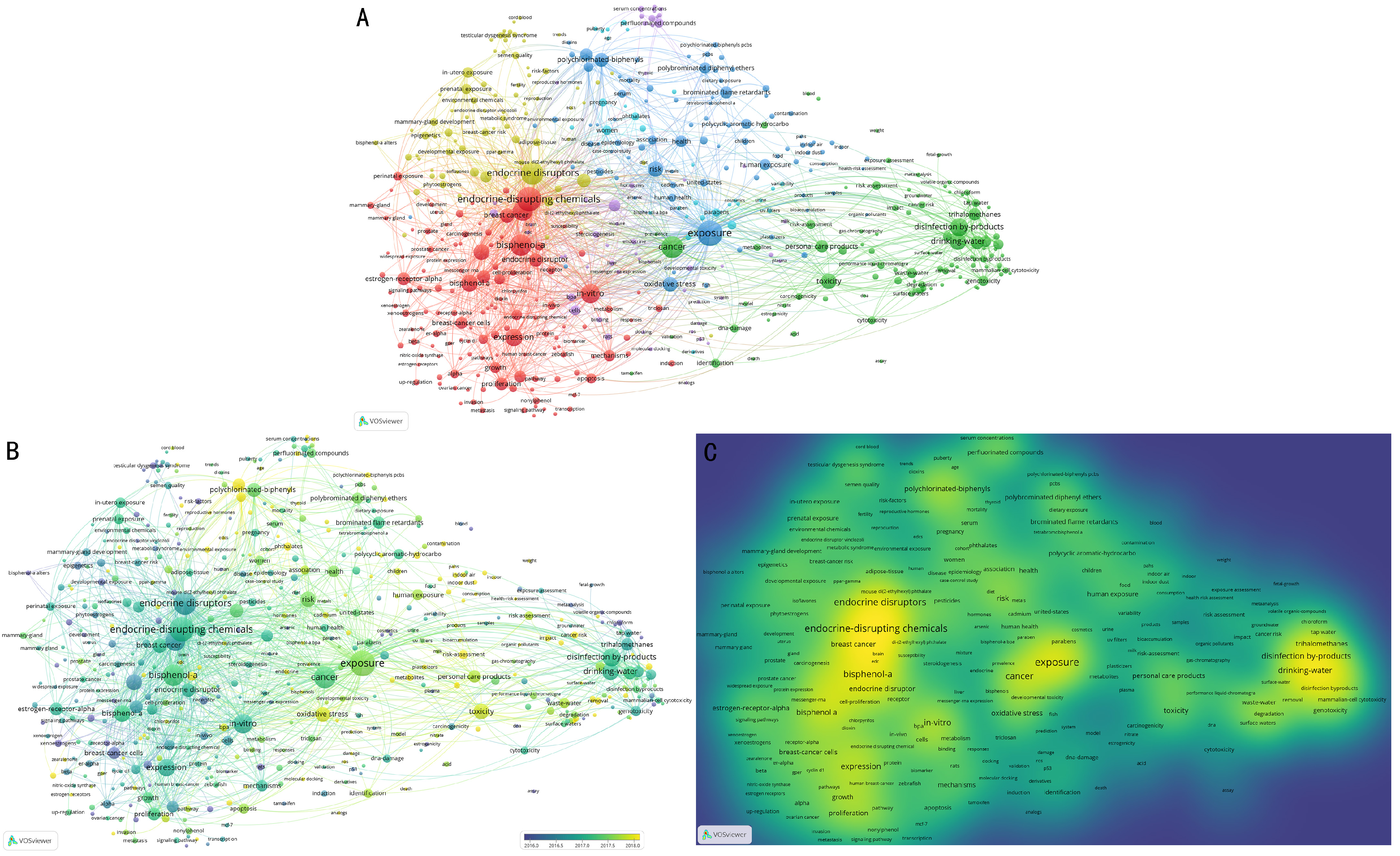


**Supplementary Figure S5** Co-occurrence analysis of keywords. (A) Network visualization map of keywords of related studies (7 clusters). (B) Overlay visualization map of keywords according to average publication year (blue means earlier, yellow means later). (C) Density visualization map of keywords according to the frequency of appearance (yellow means more frequent).


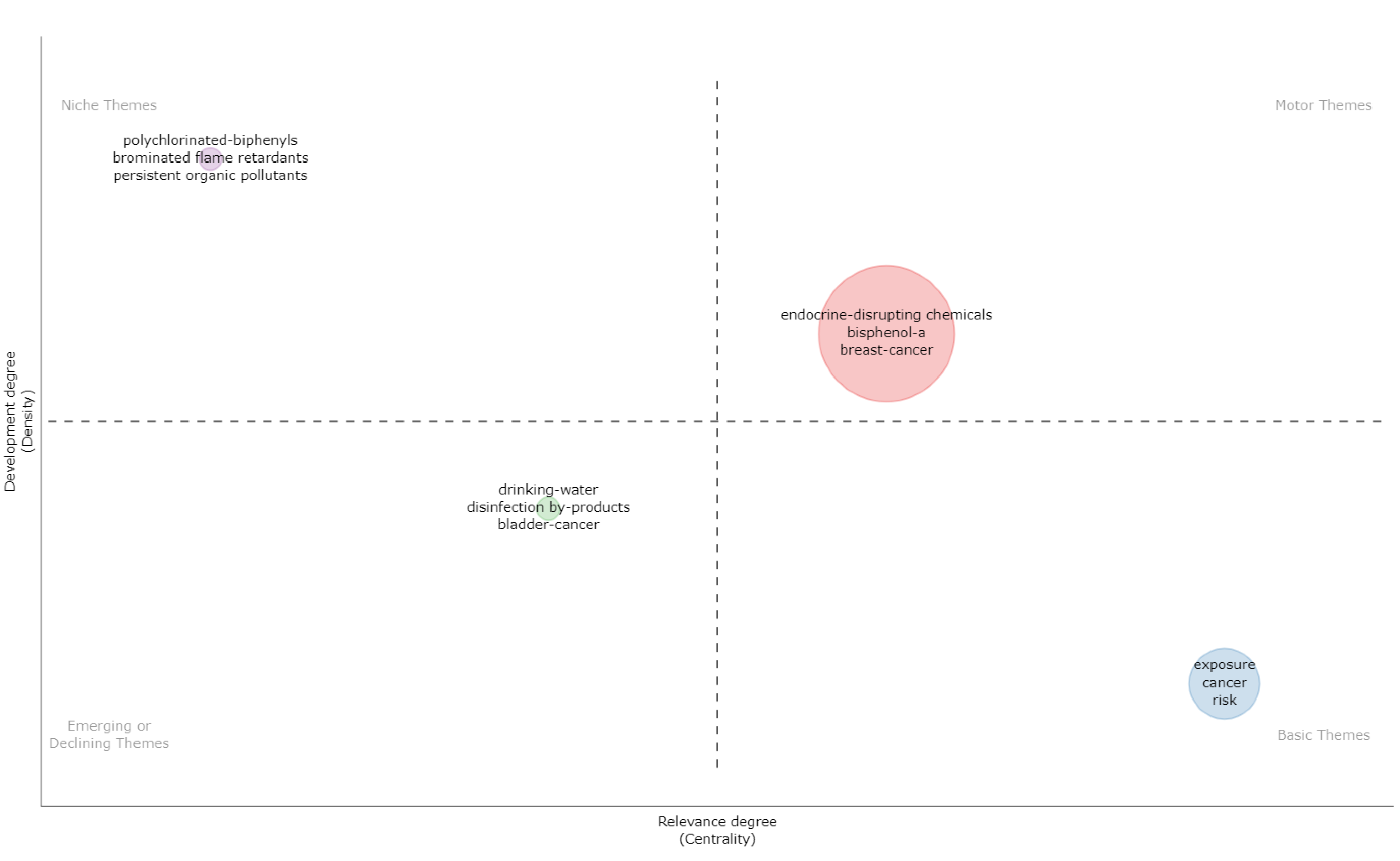


**Supplementary Figure S6** Keywords plus of four quadrants. (a) motor themes (first quadrant); (b) niche themes (second quadrant); (c) emerging or declining themes (third quadrant); (d) basic themes (fourth quadrant).


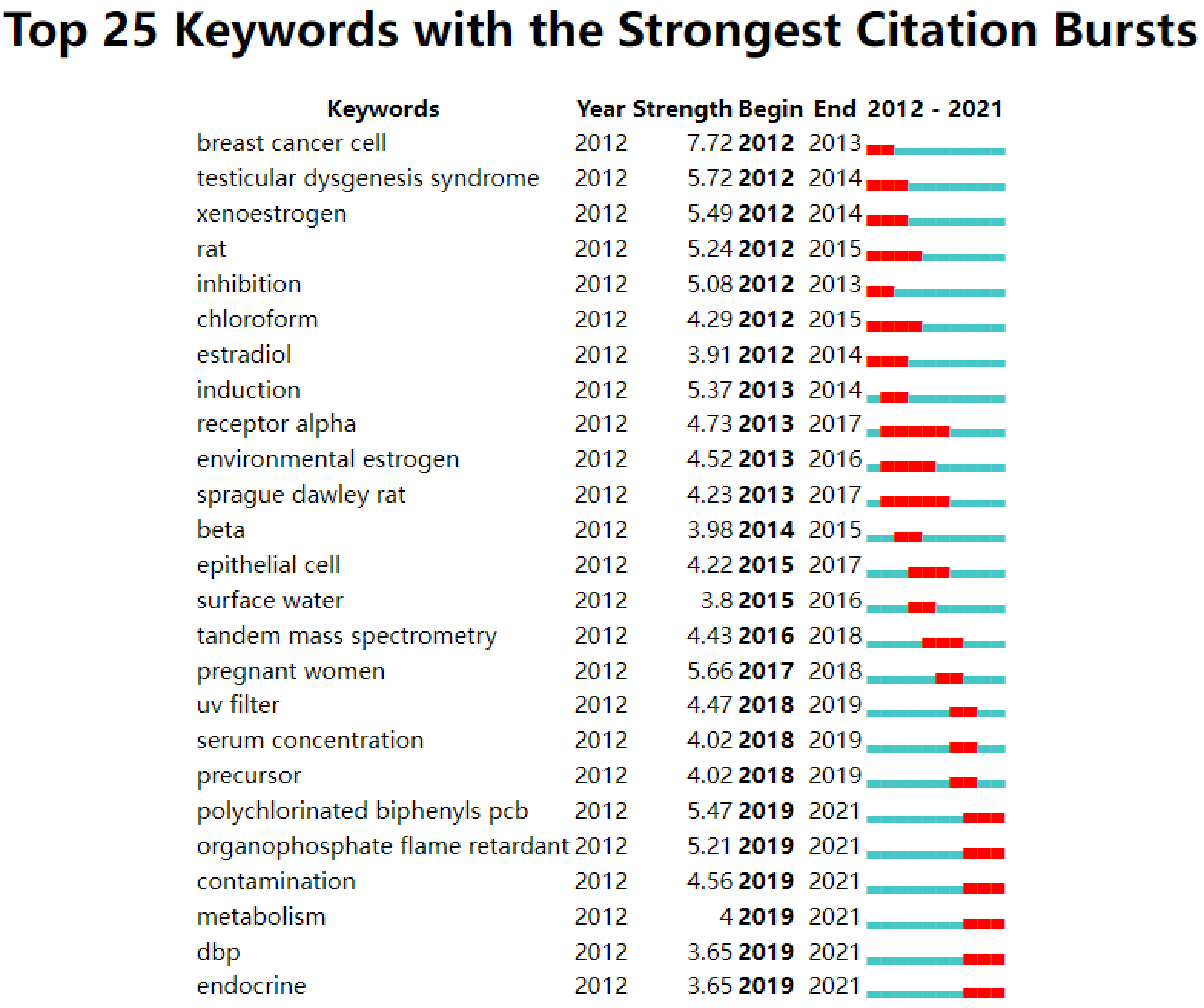


**Supplementary Figure S7** Top 25 keywords with the strongest citations bursts of this research field.
